# Surgical resection of a ruptured transverse pancreatic artery aneurysm

**DOI:** 10.1186/s40792-021-01128-4

**Published:** 2021-02-22

**Authors:** Toru Takematsu, Keisuke Kosumi, Takuya Tajiri, Kosuke Kanemitsu, Kosuke Mima, Mitsuhiro Inoue, Takao Mizumoto, Tatsuo Kubota, Nobutomo Miyanari, Hideo Baba

**Affiliations:** 1 Department of Surgery, National Hospital Organization Kumamoto Medical Center, 1-5 Ninomaru, Chuo-ku, Kumamoto, 860-0008 Japan; 2 Department of Gastroenterological Surgery, Graduate School of Life Sciences, Kumamoto University, 1-1-1 Honjo, Chuo-ku, Kumamoto, 860-8556 Japan

**Keywords:** Pancreas, Transverse pancreatic artery, Visceral artery aneurysm

## Abstract

**Background:**

Visceral artery aneurysms are rare, but they may cause heavy bleeding and high mortality. In addition, aneurysms originating from the superior mesenteric artery (SMA) account for only 1% of visceral artery aneurysms. We report the rare case of a ruptured transverse pancreatic artery aneurysm originating from the SMA that required urgent surgical treatment.

**Case presentation:**

A 66-year-old woman presented with acute back pain after lunch, and she was transported by ambulance. She had upper quadrant spontaneous pain and moderate tenderness, but no guarding or rebound pain. She had rheumatoid arthritis, and was taking 10 mg of steroids per day. Contrast-enhanced computed tomography demonstrated a retroperitoneal hematoma spreading to the ventral side of the left kidney and extravasation of contrast agent from a branch of the SMA. We diagnosed rupture of aneurysm. We conferred with our IVR team on treatment strategy for the ruptured aneurysm. In addition, we finally selected operation, since the branch of the SMA to the aneurysm was too thin and complex to conduct IVR. For this reason, we performed emergency simple aneurysmectomy of the transverse pancreatic artery. The postoperative course was relatively smooth.

**Conclusion:**

Rupture of a transverse pancreatic artery aneurysm originating from the SMA is rare. However, when diagnosing patients with acute abdomen or back pain, we should consider rupture of a visceral artery aneurysm. Endovascular treatment may currently be common for ruptured visceral artery aneurysms, but we should flexibly treat them according to the patient’s condition and facility considerations.

## Background

Visceral artery aneurysms are rare, but rupture can be life threatening [1]. Of patients with visceral artery aneurysms, 22% are diagnosed after rupture, resulting in misdiagnosis due to different clinical conditions and high mortality rates of 8.5–25% [2–4]. Aneurysms originating from the superior mesenteric artery (SMA) account for only 1% of visceral artery aneurysms and their diagnosis is markedly difficult.

The transverse pancreatic artery (TPA) is found in approximately 60–70% [5]. The TPA generally branches from the dorsal pancreatic artery, which originates from the splenic artery, the common hepatic artery, the SMA, and the celiac artery, or directly from the splenic or SMA [5–8]. Only approximately 2.5% of the TPA directly branches from the SMA and supplies blood to the underside of the pancreas [6, 9]. We report a case of a ruptured aneurysm of the TPA originating from the SMA that required urgent surgical treatment.

## Case presentation

A 66-year-old woman presented with acute back pain after having lunch and she was transported by ambulance. Her body temperature was 35.9 °C, heart rate was 80 bpm, blood pressure was 101/65 mmHg, and consciousness was clear. On abdominal examination, she had upper quadrant spontaneous pain and moderate tenderness, but no guarding or rebound pain. She had a duodenal ulcer several years ago. She was taking 10 mg of steroids per day for rheumatoid arthritis.

**Fig. 1:**
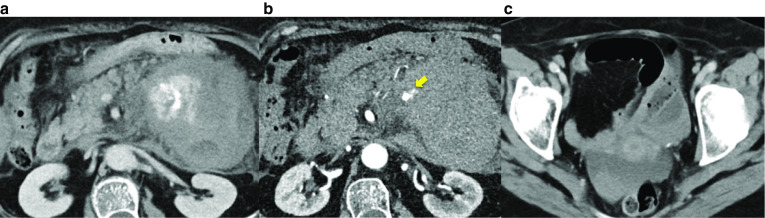
Contrast enhanced computed tomography. **a** The retroperitoneal hematoma and extravasation of contrast spread to the ventral side of the left kidney. **b** Aneurysm (yellow arrow) from a branch of the superior mesenteric artery. **c** Ascites in the pelvis

**Fig. 2:**
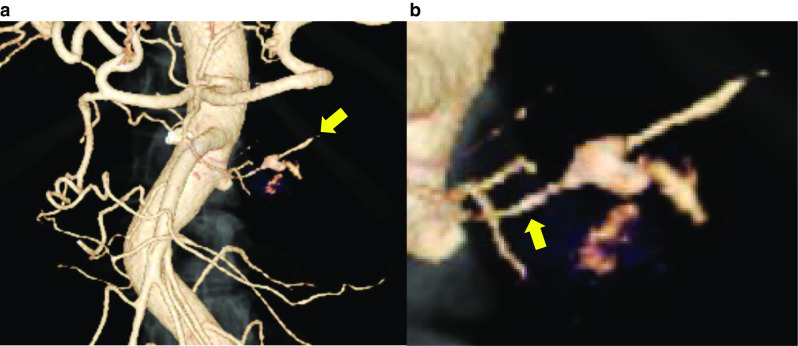
Three-dimensional computed tomography angiography showing a transverse pancreatic artery aneurysm from the superior mesenteric artery (SMA). **a** Celiac artery and SMA branching from the aorta. An aneurysm (yellow arrow) is observed on the left side of the SMA. **b** An enlarged image of the aneurysm. It branched from the SMA (yellow arrow)

The admission laboratory data included the following: white blood cell count, 18.2 × 10^3^/μl, hemoglobin, 10.7 g/dl, hematocrit, 32.4%, platelets, 22.6 × 10^4^/μl, C-reactive protein, 3.32 mg/dl, aspartate aminotransferase, 25 IU/l, alanine aminotransferase, 49 IU/l, and serum amylase, 43 IU/l. Contrast-enhanced computed tomography (CT) demonstrated a retroperitoneal hematoma at the ventral side of the left kidney, extravasation of contrast agent from a branch of the SMA, and ascites in the pelvis (Fig. 1a–c). Utilizing detailed three-dimensional CT (3DCT), we confirmed the extravasation of contrast agent from a branch of the SMA (Fig. 2a and b).

**Fig. 3:**
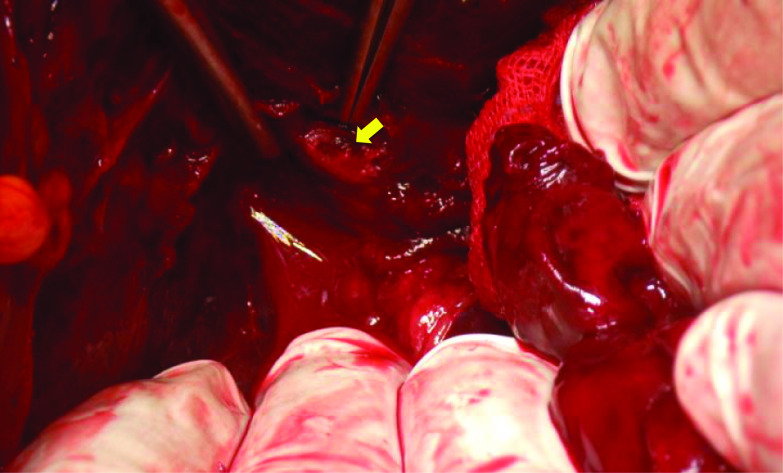
Intraoperative photography of the aneurysm (yellow arrow)

We diagnosed her with a ruptured artery aneurysm of the TPA originating from the SMA. We conferred with our IVR team on treatment strategy for the ruptured aneurysm. In addition, we finally selected operation since the branch of the SMA to the aneurysm was too thin and complex to conduct IVR. In addition, the patient went into shock in the emergency room. For these reasons, we immediately performed emergency surgery. When we opened the abdomen, we found hematoma in the omental bursa. We opened the omental bursa and removed the transverse mesocolon from the lower edge of the pancreas. There was massive hematoma behind the pancreas. After removing the hematoma, we found that a branch of the SMA was bleeding. This branch was the TPA and it had a hemorrhagic aneurysm of approximately 5 mm (Fig. 3). We ligated and resected the aneurysm, and the surgery was finished. The patient was hospitalized in the intensive care unit for 3 days. Although she had pancreatitis, the postoperative course was relatively smooth and she was discharged home 30 days after surgery.

## Discussion

Visceral aneurysms are rare, but when limited to peripancreatic aneurysms, most of those aneurysms originate from the gastroduodenal and pancreatic duodenal arteries [10]. In our case, we observe the ruptured aneurysm of TPA originating from the SMA. An aneurysm in the SMA is rare, and its etiology is unclear. One-third of SMA aneurysms are due to septic embolism [11]. Other etiologies include arteriosclerosis, polyarteritis, nodosa, pancreatitis, biliary tract disease, neurofibromatosis, and trauma [11]. In our case, the history of rheumatoid arthritis may have played a role in aneurysm formation.

The TPA generally branches from the dorsal pancreatic artery, and runs behind the pancreas [6, 7]. Therefore, aneurysm rupture at the TPA causes bleeding in the retroperitoneum. In the case of bleeding from the aneurysm at the head of the pancreas, hemorrhage may be excreted to the pancreatic duct, bile duct, or gastroduodenum, and the symptoms may resemble those of biliary-pancreatic disease [12–15]. In the case of the TPA aneurysm, it found with the onset of pancreatitis have been reported [16]. However, bleeding of the caudal pancreas, as in our case, does not cause such symptoms or an increase in pancreatic enzymes.

Our patient presented with acute back and abdominal pain, but her vital signs were first stable. Her history of a duodenal ulcer several years ago and current administration of 10 mg of steroids per day may have delayed the diagnosis of a ruptured TPA aneurysm, leading to heavy bleeding.

Abdominal contrast-enhanced CT is useful for finding extravasation. Selective angiography of abdominal artery enables us to find the site of bleeding in greater detail and treat aneurysms at the same time.

When it comes to treating TPA, it was reported that a TPA aneurysm which was successfully embolized by angiography in 1982 [16]. In this case report, there is one week between diagnosis and treatment. This patient had time to spare because the aneurysm had not ruptured. In our case, the patient went into shock in the emergency room. In addition, the branch of the SMA to the aneurysm was too thin and complex to conduct IVR. Endovascular treatment could pose a risk of covering important branches of the SMA main trunk and could make embolization of the distal branch difficult [17]. Therefore, we selected surgical hemostasis.

Surgical treatment can be performed by simple ligation in most cases, but sometimes more invasive procedures are required [18]. For a ruptured splenic aneurysm, splenectomy or distal pancreatectomy may be performed [19]. On the other hand, pancreaticoduodenectomy is performed for approximately 14% of ruptured pancreaticoduodenal aneurysms [13]. In our case, the aneurysm was only 5 mm, and simple ligation and resection of the aneurysm were sufficient to control hemorrhage.

In addition, the hematoma may not reveal the bleeding point and emergency laparotomy may endanger the patient’s life. However, if the bleeding point is known before surgery, the advantage of surgery is that it can stop bleeding directly. We pressed the pancreas itself with blocking forceps to stop the bleeding. After hemostasis, we found a TPA aneurysm and ligated it. Postoperative pancreatitis may be caused by pressure on the pancreas during the operation, but it does not have a significant impact on the postoperative course.

## Conclusion

Rupture of a TPA aneurysm originating from the SMA is rare. However, when diagnosing patients with acute abdomen or back pain, we should consider rupture of a visceral artery aneurysm. Patients with ruptured visceral aneurysms should be flexibly treated according to their condition and facility.

## Data Availability

All data generated or analyzed during this study are included in this published article.

## Abbreviations

CT: Computed tomography
SMA: Superior mesenteric artery
TPA: Transverse pancreatic artery
3DCT: Three-dimensional computed tomography
